# The effects of dexmedetomidine and ketamine infusions on the inflammatory response in liver resection: A randomized double-blind placebo study

**DOI:** 10.1097/MD.0000000000042999

**Published:** 2025-07-04

**Authors:** İrem Ates, Esra Laloglu, Salih Kara, Tuba Yaman, Bahar Isik

**Affiliations:** a Department of Anesthesiology and Reanimation, Faculty of Medicine, Ataturk University, Erzurum, Turkey; b Department of Medical Biochemistry, Faculty of Medicine, Ataturk University, Erzurum, Turkey; c Department of Transplantation Center, Faculty of Medicine, Ataturk University, Erzurum, Turkey; d Department of Emergency, Faculty of Medicine, Binali Yildirim University, Erzincan, Turkey.

**Keywords:** dexmedetomidine, inflammation, infusion, ketamine, liver resection, pain

## Abstract

**Background:**

This study compared the effects of ketamine and dexmedetomidine (Dex) on inflammation and pain in liver resection surgery.

**Methods:**

Forty-five American Society of Anesthesiologists class III patients aged 18 to 65 scheduled for liver resection surgery were randomized into 3 equal groups. The ketamine group received an intravenous ketamine bolus (0.5 mg/kg) during anesthesia induction and continuous low-dose infusion at 0.25 mg/kg/hour. In the Dex group, intravenous infusion was initiated at a 1 µg/kg bolus for the first 10 minutes, and at 0.5 µg/kg/hour after intubation. The control group patients were infused crystalloid solution at 8 mL/kg/hour from induction. Venous blood was collected at postoperative hours 1 and 12 for pentraxin 3, serum amyloid A, hepcidin, and inflammatory marker analysis. Visual analogue scale (VAS) values were recorded.

**Results:**

Pentraxin 3, serum amyloid A, and hepcidin continued to rise at 12 hours in the control group, but began declining in the Dex and ketamine infusion groups (*P* < .05). VAS levels and fentanyl consumption decreased in the ketamine and Dex groups compared to the control group (*P* < .05). The decreases in inflammatory parameters, VAS scores, and fentanyl consumption were similar between the ketamine and Dex groups (*P* > .05). A positive correlation was observed between inflammation levels and pain severity (*P* < .001). There was no difference in liver function tests between any of the groups (*P* < .05).

**Conclusion:**

Ketamine and Dex infusions were both effective in reducing inflammation and pain following liver resection, with no obvious superiority of one over the other.

## 1. Introduction

Liver resection is recognized as the most effective option in the treatment of both primary and metastatic hepatic tumors. Ischemia–reperfusion injury (IRI) is the principal underlying cause of regenerative failure, and develops following a decreased blood flow to an organ (the Pringle maneuver) and the subsequent restoration of blood flow to ischemic tissues, which can further exacerbate liver failure. Cytokines such as interleukin-1 (IL-1), IL-6, tumor necrosis factor (TNF-α) and interferon gamma released into the environment with the reperfusion of ischemic tissues can affect several organs, particularly the liver, by leading to inflammation. Despite being the focus of scientific interest for many years, and although numerous pharmacological and surgical treatment interventions have been investigated, IRI remains problematic in liver resections. Reducing inflammation deriving from IRI may therefore represent a potential therapeutic objective for the alleviation of clinical problems observed following hepatic resection^.[1–4]^

Ketamine and dexmedetomidine (Dex), are sedative, analgesic, and anesthetic agents frequently employed in anesthesia. Ketamine is a phencyclidine derivative that entered into clinical use in the 1960s.^[5]^ It is an N-methyl-d-aspartate receptor antagonist that exhibits an analgesic effect at the spinal cord level. However, ketamine use is limited due to hallucinations and agitation during waking. However, it exhibits a potent analgesic effect when used at subanesthetic doses, and psychotomimetic side-effects at these doses are rare.^[6]^ Recent studies have also investigated the anti-inflammatory effects of ketamine.^[7,8]^

Dex is a highly selective α2-adrenergic receptor agonist. It is known to possess anxiolytic, sedative, analgesic, sympatholytic, and anti-inflammatory characteristics and to cause only minimal respiratory depression, and can be administered via a number of different routes.^[9]^ These qualities make it safe and versatile. Dex exhibits an immunomodulatory and organ-protecting effect by inhibiting the release of pro-inflammatory cytokines and caspase-3 activation. It also produces analgesia via decreased substance P and glutamate release and interneuron hyperpolarization.^[10,11]^

Studies have demonstrated the potent anti-inflammatory and analgesic effects of these 2 agents on an individual basis.^[7,12–15]^ There are therefore strong indications that giving Dex plus ketamine to patients undergoing liver resection surgery may reduce post-procedural inflammation and pain. The aim of this study was to evaluate and compare the effects of ketamine and Dex on inflammation and postoperative pain in liver resection surgery.

## 2. Materials and methods

The randomized, prospective, double-blind study was conducted in accordance with the Declaration of Helsinki, and the protocol was approved by the Local Ethics Committee of Ataturk University (no. B.30.2ATS.0.01.00/625) on [June 30, 2022]. All the patients taking part received detailed information about the study procedures, and written informed consent was obtained. The study was registered on ClinicalTrials.gov (Registration Number: NCT06219928).

Forty-five American Society of Anesthesiologists class III patients aged 18 to 65 and scheduled for liver resection surgery were included in the study. In addition, since the severity of hepatocyte damage may vary, operations with similar ischemia durations were included. American Society of Anesthesiologists class IV patients, those with known cardiovascular, renal, or hematological diseases, psychiatric disorders, with allergies to one of the drugs to be used, or who were unable to establish communication, and pregnant women were excluded. Fifty-nine patients were originally included in the study. However, 12 did not meet the inclusion criteria and 2 declined to participate, and the research was concluded with 45 patients.

The 45 patients were randomly assigned into one of 3 groups of 15 members each using computer software, a control group, a low-dose ketamine infusion group, and a Dex infusion group. The practitioner, patient, and postoperative pain assessors were blinded to which group received which drug. Saline preparations for the control group involved identical volumes of preoperative and intraoperative infusion solutions to those used for the other groups in order to establish double-blinding.

### 2.1. Study design

Infusion with 0.9% NaCl was initiated prior to anesthesia induction. Preoxygenation was performed using 100% O₂, and propofol (2–3 mg/kg), fentanyl (2 μcg/kg), and rocuronium (0.6 mg/kg) were administered for anesthesia induction. Oral intubation was performed in all cases. Anesthesia was maintained by means of 1% to 2% sevoflurane (2 L fresh gas flow) and a 50% oxygen and 50% air mixture. Crystalloid infusion (8 mL/kg/h) was maintained throughout the procedure. Depth of anesthesia was monitored using the Bispectral Index, values being kept between 40 and 60.

The control group (n = 15) received a bolus dose of crystalloid during induction for the first 10 minutes, followed by crystalloid infusion solution at 8 mL/kg/hour after intubation.

In the ketamine group (n = 15), the patients received an intravenous (iv.) ketamine (Ketax, İstanbul, Turkey) bolus (0.5 mg/kg) during induction for the first 10 minutes, and continuous low-dose infusion at 0.25 mg/kg/hour following intubation.

In the Dex group (n = 15), the patients first received an iv. Dex (Semotidine, İstanbul, Turkey) bolus of 1 µg/kg for 10 minutes during induction of anesthesia, while infusion at 0.5 µg/kg/hour was initiated following intubation.

After being prepared at a pharmacy, each syringe was coded and labeled. A 20-mL syringe diluted with crystalloid was used to provide the groups’ bolus dosage. This bolus dose is administered directly. Fifty-milliliter injectors diluted with crystalloid were used for all infusions, they were performed till using an infusion pump. Continuous infusions were stopped at the end of the first hour postoperatively.

All patients underwent standard electrocardiography, peripheral oxygen saturation (SpO_2_), and noninvasive blood pressure monitoring. All values were recorded at 5-minute intervals intraoperatively, and crystalloid infusion (8 mL/kg/hour) was maintained during the operations.

### 2.2. Surgical technique

Liver resections were performed by the same surgical team using the same technique in all cases. Resection was applied to patients with metastatic liver disease (23), primary hepatic tumors (n = 10), and alveolar echinococcosis (n = 12). Prior to surgery, the patients were evaluated according to their Eastern Cooperative Oncology Group (ECOG) performance status (ECOG 0 = the patient is asymptomatic, ECOG 4 = the patient is bedridden). Patients with ECOG performance scores of 0, 1, or 2 were regarded as suitable for surgery. The patients underwent major hepatectomy and segmentectomy in which at least 3 segments were removed. The Pringle maneuver was applied for bleeding control during parenchymal transection in the intraoperative period. Parenchymal transection was performed using bipolar cautery, coagulating and cutting energy devices, and ultrasonographic aspirators. Patients were observed in the intensive care unit for 1 to 2 days postoperatively and then transferred to the normal ward.

### 2.3. Postoperative analgesia management

The same postoperative analgesia management protocol was applied to all 3 study groups. All patients received 1000 mg iv. paracetamol (Perfalgan 10 mg/mL, Bristol-Myers Squibb, France) 30 minutes prior to the end of surgery. This was subsequently repeated every 6 hours postoperatively. Postoperative evaluation was carried out by an anesthesiologist blinded to the drugs employed for analgesia and to the study groups. Postoperative analgesia at 4 and 12 hours by means of a visual analogue scale (VAS) (VAS 0 = no pain, VAS 10 = the most severe pain). Patients registering VAS scores ≥ 4 were administered 1 mg/kg tramadol as a rescue analgesic.

### 2.4. Biochemical analysis

Blood specimens collected at postoperative hours 1 and 12 for pentraxin 3 (PTX3), serum amyloid A (SAA), and hepcidin analysis were centrifuged for 15 minutes at 4000 rpm. The resulting sera were stored at −80 °C until the day of analysis and were studied using the enzyme-linked immunosorbent analysis method.

Complete blood count values routinely investigated at the specified time periods, the inflammatory markers C-reactive protein (CRP) and IL-6 and the hepatocellular enzymes alanine aminotransferase (ALT), aspartate aminotransferase (AST), alkaline phosphatase, gamma glutamil transferase, lactate dehydrogenase, and bilirubin values were recorded for all patients.

### 2.5. Statistical analysis

Data in the “Effect of Oxycodone hydrochloride combined with Dex on quality of recovery and stress response after general anesthesia in patients who had Laparoscopic Cholecystectomy”^[15]^ study were used in sample calculation performed on G Power 3.1.97 software (Franz Faul, Germany), with an assumed effect size of d: 0.692. Calculation performed with that effect size, 95% power, and a 5% margin of error revealed that a sample of size of 36 would be required, with a minimum of 12 in each group.

Data analysis was conducted on SPSS version 25.0 software. The variables’ compatibility with normal distribution was examined using histogram charts and the Kolmogorov–Smirnov test. Mean, standard deviation, median, and minimum–maximum values were used for descriptive analyses. The Kruskal–Wallis test was applied to evaluate nonparametric variables between more than 2 groups. Measurement data were compared using Spearman correlation test. *P* values < .05 were considered statistically significant.

## 3. Results

**Figure 1.**
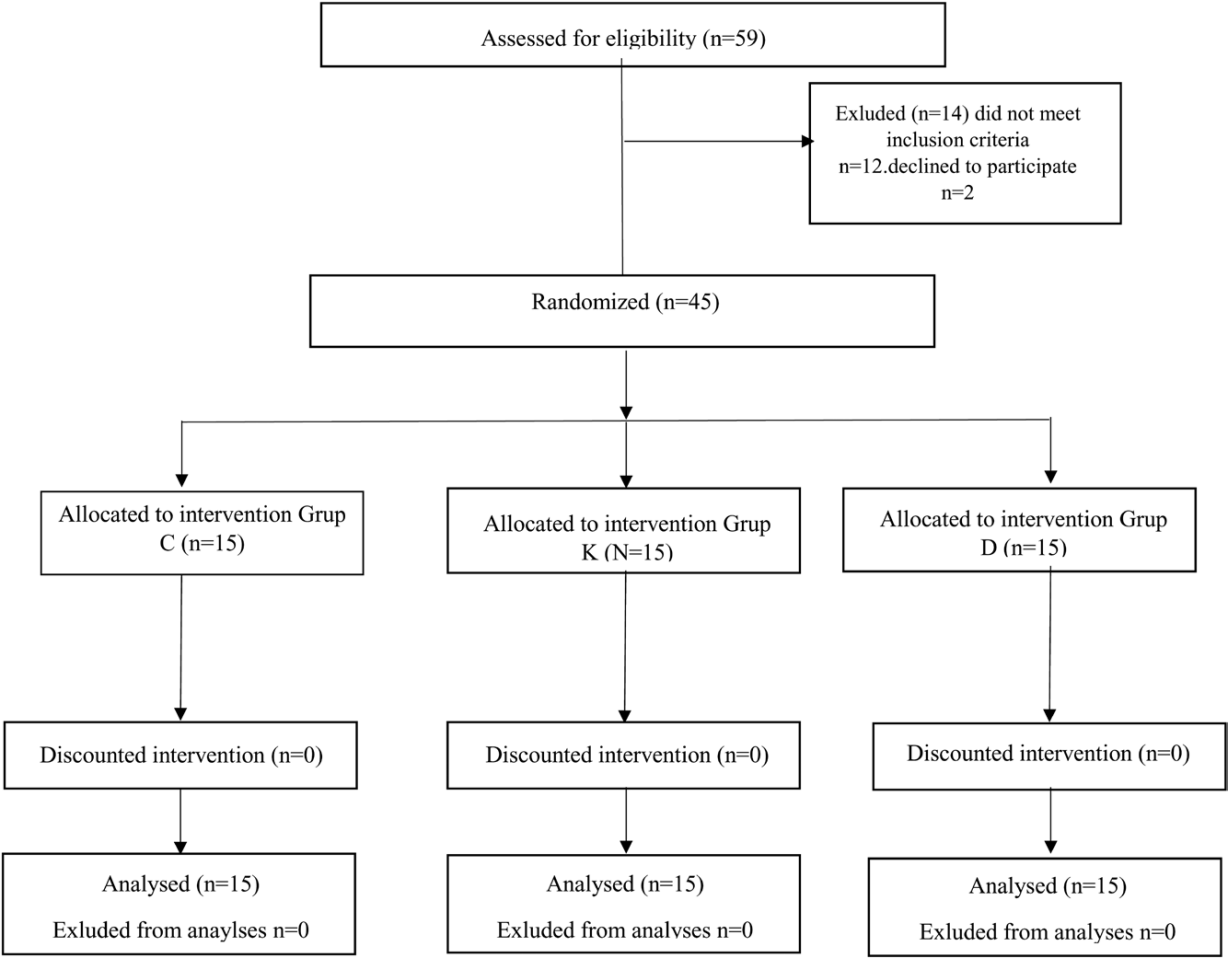
Consolidated standards of reporting trials flow diagram. CONSORT diagram. CONSORT indicates consolidated standards of reporting trials.

No significant differences were determined between the groups in terms of sex, age, or body mass index. The enrollment and allocation of the patients are shown in the CONSORT diagram (Fig. 1). The groups’ operative times were also similar. However, control group VAS scores and fentanyl consumption at hours 4 and 12 were significantly higher than those in the ketamine and Dex groups. However, VAS scores and fentanyl consumption were similar between the ketamine and Dex groups. Additional opioid analgesic requirements were significantly higher in the control group than in the ketamine and Dex groups (*P* = .01 and *P* = .025, respectively). However, there were no significant differences found between the ketamine and Dex groups (*P* = .705). Surgery and anesthesia times were similar (Table 1).

**Table 1:** The patients’ demographic and clinical data.

		Control group (n = 15)	Ketamine group (n = 15)	Dexmedotomidine group (n = 15)	*P*
Sex	Female	9 (60%)	5 (33.3%)	8 (53.3%)	.315
	Male	6 (40%)	10 (66.7%)	7 (46.7%)	
Age (years)		50.47 ± 15.40	58.87 ± 11.28	50.06 ± 20.00	.245
BMI (kg/m²)		25.37 ± 2.86	24.34 ± 3.28	24.14 ± 3.61	.558
Comorbidities (n)	Yes	8 (53.3%)	6 (40%)	6 (40%)	
	No	7 (46.7%)	9 (60%)	9 (60%)	
Duration of the anesthesia (minutes)		409.2 ± 52.45	376.67 ± 82.03	362.8 ± 124.21	.368
Duration of the surgery (minutes)		367 (243–448)	348 (212–523)	342 (95–3328)	.669
VAS 4th hour		6 (5–9)	4 (2–6)	4 (2–5)	<.001*^,^† .116‡
VAS 12th hour		5 (3–8)	3 (2–5)	4 (1–6)	<.001* .008† .775‡
Postoperatif fentanyl consumption (µg)		775 (450–900)	380 (75–550)	450 (150–650)	<.001*^,^† .595‡
Rescue analgesia (yes/no)		3/12	5/10	6/9	.01* .025† .705‡

The data were expressed as n (%), mean ± SD, and median (min–max) value.

n = number of patients, BMI = body mass index, VAS = visual analogue scale.

* Between the control and ketamine groups, *P* < .05: statistically significant.

† Between the control and dexmedetomidine groups, *P* < .05: statistically significant.

‡ Between the ketamine and dexmedetomidine groups, *P* < .05: statistically significant.

No significant differences were observed between the control, ketamine, and Dex groups in terms of liver function tests at postoperative hours 1 and 12. This shows that ketamine and Dex have no effect on such tests (Table 2).

**Table 2:** The groups’ liver function tests.

	Control Group (n = 15)	Ketamine group (n = 15)	Dexmedetomidine group (n = 15)	*P*
AST 1st hour	387 (45–1987)	355 (79–1004.7)	284.7 (105.1–1410.4)	.936
AST 12th hour	269 (23–1896)	280.3 (66–852.2)	267.4 (90.6–1278.7)	.767
ALT 1st hour	267 (19–1196)	278.4 (25–1262.2)	244.6 (77–1069.2)	.876
ALT 12th hour	307 (12–1208)	266.3 (31–1534.1)	298 (91.5–1235.8)	.782
LDH 1st hour	447 (150–2677)	620.3 (240–2059.6)	450 (270.4–2729.1)	.889
LDH 12th hour	350 (140–1549.2)	450.8 (189–1626.6)	409.8 (250–1745.8)	.824
Bilirubin 1st hour	1.32 (0.2–7.34)	1.98 (0.51–6.71)	2.63 (0.64–6.24)	.951
Bilirubin 12th hour	0.88 (0.19–7.28)	1.8 (0.53–6.19)	1.61 (0.53–7.53)	.444
GGT 1st hour	41.3 (12–346)	40.6 818–312.3)	84.3 (11.5–294.6)	.358
GGT 12th hour	39 (13–431)	45.6 (14–283.1)	86 (9.9–298.2)	.244

Data expressed as median (min–max) values. *P* < .05: statistically significant.

AST = aspartate aminotransferase, GGT = gamma glutamyl transferase, LDH = lactate dehydrogenase, T. Bilirubin.

Inflammatory parameters such as SAA, hepcidin, PTX3, CRP, and IL-6 were higher in the control group at the first hour than in the ketamine and Dex groups. By the 12 hour, however, while these parameters continued to rise in the control group, a decrease had commenced in both the ketamine and Dex groups. Inflammatory parameters in the ketamine and Dex groups were similar to one another at postoperative hours 1 and 12 (Table 3).

**Table 3:** The effects of ketamine and dexmedetomidine use on inflammatory parameters.

	Control group (n = 15)	Ketamine group (n = 15)	Dexmedetomidine group (n = 15)	*P*
SAA 1st hour	7928.28 (6692.36–8423.58)	7096.83 (3407.16–8013.58)	6641.83 (3052.16–7673.58)	<.001*^,^† .305‡
SAA 12th hour	9428.28 (7637.36–9923.58)	6032.84 (2319.24–6833.14)	5735.43 (1964.24–6493.14)	<.001*^,^† .305‡
Hepcidin 1st hour	80.52 (62.48–93.15)	68.24 (37.4–77.15)	63.41 (21.18–75.21)	<.001*^,^† .174‡
Hepcidin 12th hour	94.25 (65.88–110.45)	50.9 (6.15–61.96)	45.43 (10.86–57.64)	<.001*^,^† .325‡
PTX3 1st hour	25.08 ± 3.88	18.51 ± 4.02	16.81 ± 3.34	<.001*^,^† .437‡
PTX3 12th hour	36.79 ± 2.06	8.86 ± 2.16	8.23 ± 2.24	<.001*^,^† .708‡
CRP 1st hour	26.9 (16.36–74.3)	18.65 (9.87–38.8)	18.80 (9.50–45.24)	.013* .016† .775‡
CRP 12th hour	53.69 (21.72–126.64)	10.65 (1.43–29.8)	10.80 (1.50–37.24)	<.001*^,^† .935‡
IL-6 1st hour	238.04 ± 139.82	179.39 ± 58.76	171.00 ± 62.71	.025* .039† .651‡
IL-6 12th hour	263.40 ± 158.28	139.23 ± 59.37	148.61 ± 55.45	.010* .006† .486‡

Data expressed as (%), mean ± SD and median (min–max) values.

CRP = C-reactive protein, IL-6 = interleukin 6, PTX3 = pentraxin 3, SAA = serum amyloid A.

* Between the control and ketamine groups, *P* < .05: statistically significant.

† Between the control and dexmedetomidine groups, *P* < .05: statistically significant.

‡ Between the ketamine and dexmedetomidine groups, *P* < .05: statistically significant.

When the entire patient group was evaluated, pain severity increased in line with inflammation marker levels. A powerful, significant positive correlation was determined between inflammatory parameters at the first hour and VAS scores at the fourth hour (*P* < .001). Similarly, a powerful, significant positive correlation was observed between these parameters at 12 hours and VAS scores at 12 hours (*P* < .001) (Table 4).

**Table 4:** Correlations between inflammatory parameters and VAS scores.

		VAS 4th hour	VAS 12th hour
SAA 1st hour	*r*	0.860	0.494
	*P*	<.001	<.001
SAA 12th hours	*r*	0.703	0.837
	*P*	<.001	<.001
Hepcidin 1st hour	*r*	0.807	0.720
	*P*	<.001	<.001
Hepsicidin12th hour	*r*	0.736	0.791
	*P*	<.001	<.001
PTX3 1st hour	*r*	0.840	0.471
	*P*	<.001	<.001
P TX3 12th hour	*r*	0.753	0.859
	*P*	<.001	<.001
CRP 1st hour	*r*	0.806	0.352
	*P*	<.0011	.018
CRP 12th hour	*r*	0.743	0.901
	*P*	<.001	<.001
IL-6 1st hour	*r*	0.845	0.455
	*P*	<.001	<.001
IL-6 12th hour	*r*	0.633	0.864
	*P*	<.001	<.001

CRP = C-reactive protein, IL-6 = interleukin 6, PTX3 = pentraxin 3, SAA = serum amyloid A, VAS = visual analogue scale.

*P* < .05: statistically significant, *r*: correlation coefficient.

Known side-effects of ketamine (hallucinations, nausea, vomiting, hypertension, and tachycardia) and Dex (bradycardia and hypotension) were not observed in any patient.

## 4. Discussion

This study evaluated and compared the effects of low-dose ketamine and Dex infusion on postoperative inflammation and pain during liver resection. Both Dex and low-dose ketamine infusions in the perioperative period were found to lead to a significant reduction in IRI-related inflammation and postoperative pain, with neither being superior to the other.

Surgical procedures are a form of trauma and can lead to the release of stress hormones and inflammatory mediators and to immune cell function impairment. This inflammatory response can result in an increased disposition to postoperative infection, delayed wound healing, and multiple organ function disorder.^[16,17]^ Increased inflammation in the perioperative period due to IRI in liver trauma, resection, and transplantation operations may further exacerbate this situation.^[18]^

In addition to its anesthetic property, ketamine is also frequently used for its positive effects on depression, and chronic and postoperative pain. Its anesthetic or analgesic effect is dose-dependent. Administered at low doses, ketamine provides good postoperative analgesia with minimal side-effects and reduces opioid consumption.^[19]^ In agreement with the previous literature, ketamine was applied at an iv. 0.5 mg/kg bolus during induction followed by 0.25 mg/kg/hour.^[14–20]^ Dex is widely used for sedation in the intensive care unit. Studies involving its analgesic and neuroprotective effects and impacts on perioperative stress, inflammation, and immune function have become increasingly common in recent years.^[14,21]^ These effects have been investigated at various drug dosages. The Dex infusion dose in the present study was based on those in previous research.^[14]^

Dex and ketamine have been reported to exert anti-inflammatory effects and enhance postoperative pain control in liver resections, thereby promoting recovery.^[22–25]^ A comprehensive meta-analysis examining perioperative Dex infusion in surgical patients revealed significant reductions in IL-6, IL-1β, TNF-α, and CRP concentrations, alongside an increase in IL-10 levels, indicating the anti-inflammatory potential of Dex.^[14]^

Consistent with our findings, a study demonstrated the protective effects of perioperative Dex infusion against IRI during liver resection, as evidenced by improved liver function tests. However, unlike our study, this research observed minimal effects of Dex on early postoperative inflammation. This discrepancy may be attributed to the use of more sensitive biomarkers in our study for early detection of inflammation.^[26]^ Similarly, Zhang et al reported that Dex administration during hepatectomy attenuated IRI-induced damage by reducing ALT and AST levels, and decreased IL-6 and TNF-α concentrations 24 hours postoperatively.^[27]^ These results align with our findings and those of Wang et al., suggesting that Dex may mitigate the inflammatory response following hepatectomy.^[28]^

Regarding ketamine, a systematic review indicated that its perioperative administration in major surgeries reduced early postoperative IL-6 concentrations, demonstrating its anti-inflammatory effects in the early postoperative period.^[29]^ Additionally, an experimental study on liver IRI showed that ketamine suppressed the production of IL-6, IL-1β, and TNF-α cytokines, indicating its potential to modulate the inflammatory response in liver injury models.^[30]^

The analgesic effectiveness of the 2 agents was clearly visible in VAS scores and fentanyl consumption. Although the numbers suggest that ketamine is more effective as an analgesic, there was no statistically significant difference between the 2 agents. Similarly to the present study, research has also investigated the administration of ketamine, with its known powerful analgesic efficacy, in the form of low-dose infusion during the perioperative period.^[31]^

Dex reduces the need for opioids intraoperatively, while ketamine reduces the need postoperatively, according to a study that examined the effects of perioperative ketamine and Dex infusions for analgesia in bariatric surgery. Notably, none of the patients in that trial experienced any significant postoperative problems, which is consistent with our own findings.^[32]^

Following pericapsular nerve group block, Dhanya et al administered intraoperative ketamine and Dex infusion for postoperative analgesia after breast surgery. They reported superior analgesia and later rescue analgesia requirements among the patients who received ketamine compared to those given Dex.^[33]^

Levels of acute phase reactants (APRs) such as CRP, IL-6, hepcidin, SAA, and PTX3 were employed to determine the effects of Dex and low-dose ketamine infusions on early period inflammation in liver section in this study. APRs are proteins largely secreted by the liver in response to physiological homeostasis disturbance as a result of events such as tissue damage, inflammation, and infection.^[34]^ Since hepatocytes produce CRP in response to pro-inflammatory mediators, particularly IL-6, as a reaction to inflammation, these parameters were used in the present study in order to evaluate the increased inflammatory response due to IRI. IL-6 and CRP values, the bases of the inflammatory response in patients, being lower in the ketamine and Dex groups than in the control group at the first hour, and this decrease persisting at 12 hours, appears to confirm the positive effects of ketamine and Dex infusions on inflammation.

Hepcidin, another APR, plays a role in the regulation of iron metabolism. Its expression increases through the mediation of various cytokines, especially IL-6, as a response to inflammation and infection. SAA is a useful marker in the evaluation of inflammation activity and in observing the effectiveness of medical treatment. Although SAA and CRP are both APRs, SAA can yield more significant results than CRP in some states resulting in inflammation.^[35]^ Guo et al also reported that SAA can be useful in predicting microvascular invasion and early tumor recurrence in patients who undergo liver resection.^[36]^ PTX3 is a basic component of natural immunity and is a novel marker of inflammatory reactions concerning the vascular wall in particular.^[37]^ Studies have shown that PTX3 possesses anti-inflammatory and cardioprotective characteristics.^[38]^ In addition, plasma PTX3 levels are a novel biomarker for determining the severity of nonalcoholic steatohepatitis and liver fibrosis.^[39]^ The significant decreases in hepcidin, SAA, and PTX3 values with ketamine and Dex infusions compared to the control group in the present study confirm the anti-inflammatory effects of these 2 anesthetic agents.

In the mildest IRI occurring during liver resections, levels of the hepatic enzymes AST, ALT, gamma glutamil transferase, lactate dehydrogenase, and bilirubin increase in the circulation and are indicative of liver damage. In the present study, the rise in liver enzymes in the Dex and ketamine groups at hours 1 and 12 postoperatively was similar to that in the control group. These findings show that Dex and ketamine infusions during the intraoperative period entail no extra damage to the liver, and that their use is safe.

There are a number of limitations to this study, including the fact that inflammation and analgesic effects were only evaluated in the early postoperative period, and the small sample sizes. Longer-term research may uncover drug-related adverse effects, especially psychosomatic problems linked to ketamine use. Furthermore, this study procedure did not establish the long-term effects of opioid use on patients.

## 5. Conclusion

Low-dose ketamine and Dex infusions reduced inflammation and postoperative pain developing following liver resection surgery with no superiority of one over the other. We think that these agents can also be effective in improving clinical outcomes and postoperative complications after liver resection.

## Author contributions

**Conceptualization:** İrem Ates, Esra Laloglu, Salih Kara.

**Data curation:** Esra Laloglu.

**Methodology:** Esra Laloglu, Salih Kara, Bahar Isik.

**Software:** Tuba Yaman.

**Supervision:** İrem Ates, Tuba Yaman.

**Visualization:** Tuba Yaman, Bahar Isik.

**Writing – original draft:** İrem Ates.

**Writing – review & editing:** İrem Ates, Esra Laloglu, Salih Kara.
